# Combination Lorcaserin and Betahistine Treatment Improves Cognitive Dysfunction and Dopaminergic Neuron Activity in a Rat Model of Diet-Induced Obesity

**DOI:** 10.3390/brainsci15090913

**Published:** 2025-08-25

**Authors:** Ike de la Peña, Johnny Figueroa, Wei-Xing Shi

**Affiliations:** 1 Department of Pharmaceutical and Administrative Sciences, Loma Linda University School of Pharmacy, Loma Linda, CA 92350, USA; 2 Center for Health Disparities and Molecular Medicine, Department of Basic Sciences, Physiology Division, Loma Linda University School of Medicine, Loma Linda, CA 92350, USA; 3 Department of Basic Sciences, Loma Linda University School of Medicine, Loma Linda, CA 92350, USA

**Keywords:** obesity, cognition, prefrontal cortex, combination treatment, lorcaserin, betahistine

## Abstract

**Background:** Obesity is a complex disorder with both metabolic and neurocognitive consequences, including impairments in prefrontal cortex (PFC)-dependent learning and memory. Combination pharmacotherapy may offer a more effective approach for addressing obesity-induced cognitive deficits. **Objective:** This study evaluated the effects of 30-day co-administration of lorcaserin (5-HT_2_C agonist) and betahistine (H_1_ agonist/H_3_ antagonist) in reversing cognitive deficits in a diet-induced obesity (DIO) rat model. **Methods:** Male Lewis rats were subjected to DIO and administered lorcaserin (2 mg/kg) and betahistine (5 mg/kg), either alone or in combination, via intraperitoneally implanted osmotic minipumps for 30 days. Y-maze, novel object recognition, and object-in-place (OIP) tests were used to assess cognitive functions. In vivo electrophysiological recordings were employed to examine effects of the combination treatment on ventral tegmental area (VTA) dopaminergic neuron activity. **Results:** Obese Western-diet-fed rats showed lower discrimination scores in the OIP task, a behavioral test that engages PFC functions, while their performance in the Y-maze and novel object recognition tasks was similar to that of non-obese Control-diet-fed rats. Combination treatment with lorcaserin and betahistine significantly improved the OIP scores of obese rats. However, the combination treatment did not reduce body weight or obesity-associated morphometrical parameters. Electrophysiological recordings revealed a reduction in the number of spontaneously active dopaminergic neurons in the VTA of obese rats. Lorcaserin and betahistine co-treatment significantly increased the number of spontaneously active dopaminergic neurons of obese animals. **Conclusions:** These results demonstrate the potential of combination lorcaserin–betahistine treatment to reverse obesity-related cognitive deficits, possibly through enhancement of mesocortical dopaminergic neuron activity.

## 1. Introduction

Obesity, defined as a body mass index (BMI) ≥30 kg/m^2^, is a growing global health crisis [1] linked not only to metabolic and cardiovascular comorbidities but also to significant impairments in brain function [2,3]. Increasing evidence implicates chronic high-fat, Western-style diets in the disruption of neurocognitive processes, particularly those governing attention, memory, and executive function [4,5]. These obesity-related cognitive deficits are especially concerning, as they may impair dietary self-regulation and decision making, perpetuating the cycle of weight gain and poor health outcomes [4,6].

Among the brain regions most affected by obesity-induced neurochemical and structural changes is the prefrontal cortex (PFC) [3,4], a key region for higher-order cognitive functions such as working memory, behavioral flexibility, and impulse control. Preclinical studies have consistently shown that diet-induced obesity (DIO) disrupts dopaminergic signaling within the mesocortical pathway [4,5,7,8], involving PFC-projecting dopaminergic neurons originating from the ventral tegmental area (VTA). Dysfunction in this circuit has been implicated in the cognitive decline observed in both rodents and humans with obesity [4,7,9]. Notably, reductions in PFC dopamine levels have been reported in both obese humans and animals, indicating an important role for dopamine in obesity-associated cognitive impairments and dysfunctional eating behavior [4,10,11,12]. Accordingly, pharmacological strategies aimed at restoring mesocortical dopaminergic transmission have gained attention as potential treatments for the neurocognitive consequences of obesity [6,9,13].

Given the complex and multifactorial nature of obesity, combination pharmacotherapy may offer improved efficacy by targeting multiple pathways simultaneously [6,14]. We have previously proposed that lorcaserin, a selective serotonin 5-HT_2_C receptor agonist, and betahistine, a dual histamine H_1_ receptor agonist and H_3_ antagonist, could work additively or synergistically to reverse obesity-related cognitive impairments [6]. This hypothesis is based on evidence showing that activation of 5-HT2C receptors (e.g., by lorcaserin) and blockade of H3 receptors (e.g., by thioperamide, ABT-239) enhance the firing rate of dopaminergic neurons or increased dopamine levels in the PFC [15,16,17], potentially improving cognitive functions directly or indirectly via modulation of dopamine transmission. Moreover, both lorcaserin and betahistine have been shown to improve cognitive function and/or facilitate weight loss in both human and animal studies [17,18,19,20,21,22,23,24]. We proposed that lorcaserin increases the firing of mesocortical dopaminergic neurons via 5-HT_2_C receptor activation [17], while betahistine increases dopamine release by blocking inhibitory H3 receptors on dopaminergic terminals. Together, these drugs may enhance PFC dopamine levels in obese animals, thereby ameliorating cognitive dysfunction through increased dopamine receptor activation on pyramidal neurons and interneurons [6]. Of note, lorcaserin was withdrawn from the U.S. market in 2020 following concerns raised by the Food and Drug Administration regarding potential cancer risk [25]. However, given its well-characterized pharmacological profile, particularly its potency and selectivity as a 5-HT_2_C receptor agonist, we used lorcaserin as a proof-of-concept pharmacological probe to investigate how combined serotonergic and histaminergic receptor modulation influences mesocortical dopaminergic neuron function and obesity-related cognitive dysfunction.

In this study, we examined whether 30-day co-administration of lorcaserin and betahistine could reverse cognitive deficits induced by a Western diet in a DIO rat model. We hypothesized that combination lorcaserin–betahistine treatment would improve cognitive performance in obese rats. To assess cognitive function, we used Y-maze, novel object recognition, and object-in-place tasks, behavioral tests that probe memory and/or PFC-dependent processes [3,26,27]. We then used in vivo electrophysiology to examine the effects of the drug combination on dopaminergic neuron activity in the VTA. This study addresses a critical gap in the development of pharmacological interventions for obesity-related cognitive dysfunction and explores the therapeutic potential of repurposing clinically approved agents through a novel combinatorial approach.

## 2. Materials and Methods

### 2.1. Animals and Experimental Groups

Adult male Lewis rats (12 weeks old at study onset) were obtained from Charles River Laboratories (Wilmington, MA, USA) and housed in pairs under environmentally controlled conditions (12:12 h light/dark cycle; lights on at 6:00 AM, off at 6:00 PM; ambient temperature maintained at 22 ± 2 °C). Rats were fed either standard laboratory chow (13% kcal from fat; Bio-Serv Product No. F7463, Lot No. 248347) or a high-fat, Western-style obesogenic diet (41% kcal from fat; Bio-Serv Product No. F7462, Lot No. 248348) for 8 weeks. In our previous study, 8 weeks of exposure to obesogenic diet was enough to induce obesity in rats and to alter PFC-dependent functions [28,29]. Macronutrient composition and fatty acid profiles of the diets are described in prior publications [28,29,30].

A total of 80 animals (*n* = 26, control diet [CD]; *n* = 54, Western diet [WD]) were used in this study. Rats were subdivided into vehicle (CD: *n* = 14; WD: *n* = 13) and drug treatment groups (CD-lorcaserin: *n* = 12; WD-lorcaserin: *n* = 13; WD-betahistine: *n* = 13; WD-lorcaserin + betahistine: *n* = 15) while remaining on their respective diets. Obesity status was confirmed using morphometric assessments conducted after obesity induction and at the end of drug treatment, following methods described in a previous study [31]. The following indicators were recorded using laboratory weighing scales and measuring tapes: body weight and length (nose to anus length) as well as abdominal and thoracic circumferences (AC and TC, respectively). From these measurements, body mass index (BMI) was calculated as body weight (g) divided by length^2^ (cm^2^) and the Lee index as the cube root of body weight (g) divided by body length [31]. Notably, BMI was positively correlated with body fat content and served as a reliable indicator of adiposity [31]. Drug treatment groups were matched for morphometric parameters and baseline food/water intake. Only male rats were included in this study to reduce biological variability and specifically examine the neurocognitive and pharmacological effects of lorcaserin–betahistine in a DIO model. Male rodents are more prone to developing stable obesity phenotypes when exposed to high-fat diets [32,33], making them ideal models for evaluating DIO-related cognitive impairments. Additionally, excluding female rats removed potential confounding effects of estrogen and ovarian hormones, which can influence PFC function and dopaminergic neuron signaling [34,35]. While this limits generalizability to both sexes, it allowed for a more controlled investigation of serotonergic–histaminergic modulation in obesity-related cognitive dysfunction.

### 2.2. Drug Treatment

Lorcaserin hydrochloride and betahistine hydrochloride were obtained from MedChemExpress (Monmouth Junction, NJ, USA) and dissolved in 0.9% sterile saline. Following DIO induction, rats received continuous drug administration for 30 days via intraperitoneally implanted osmotic minipumps (Alzet, Cupertino, CA, USA), which minimized handling stress and ensured consistent drug delivery. Briefly, pumps were filled with a single drug solution and implanted in the animals under anesthesia. Drug release was driven by osmotic pressure, allowing for constant infusion over the treatment period without the need for repeated handling. Vehicle-treated animals received one pump containing vehicle (0.9% saline) only. For combination treatments, two separate pumps were implanted, each delivering one of the drugs, as individual pumps cannot administer multiple compounds independently. The selected doses of lorcaserin (2 mg/kg) and betahistine (5 mg/kg) were based on prior studies reporting low yet effective concentrations capable of inducing metabolic and neurophysiological changes [36,37] as well as our preliminary electrophysiological data showing modulation of dopaminergic neuron activity at these doses. The use of subthreshold doses allowed us to evaluate potential additive or synergistic effects of the combination treatment on cognitive function of animals.

### 2.3. Cognitive Behavioral Testing

Y-maze, novel object recognition (NOR), and object-in-place (OIP) tests were conducted following previously published protocols with some modifications [3,38,39,40]. Rats were habituated to the testing arena prior to behavioral evaluations. Testing was conducted in the order listed above, with a 24 h interval between each task, and assessments were performed at two points: (1) baseline (prior to drug administration) and (2) one day following completion of the 30-day drug treatment period. All behavioral testing was carried out by an experimenter blinded to treatment groups. Behavioral apparatuses were cleaned with Quatricide before each test session to eliminate olfactory cues and prevent contamination.

#### 2.3.1. Spontaneous Alternation Y-Maze Task

The Y maze consisted of three arms (each 45 cm long × 10 cm wide × 20 cm high) arranged at 120° angles. Rats were placed at the end of a designated start arm and allowed to explore freely for 8 min. The sequence and number of arm entries were recorded using an automated tracking system (Ethovision System, Noldus I.R., Wageningen, The Netherlands). Spontaneous alternation was calculated as the percentage of consecutive triplet entries into all three different arms, serving as a measure of spatial working memory [38,39].

#### 2.3.2. Novel Object Recognition (NOR) Task

The NOR task was conducted in an open-field arena (70 cm × 40 cm × 22 cm). During the familiarization phase, rats were allowed to explore two identical objects placed in opposite corners for 10 min. The following day, one familiar object was replaced with a novel object, and rats were reintroduced to the arena for a 5 min test session. Exploration time (defined as sniffing or touching within 2 cm of the object) was recorded manually by an experimenter. Exploratory time spent for novel objects was recorded, and percent exploratory preference was computed as [time in novel/(time in novel + time in old object)] in a blind manner [40].

#### 2.3.3. Object-in-Place (OIP) Task

The OIP task was performed in the same testing arena used for NOR. During the familiarization phase, rats were exposed to four different objects placed in fixed locations for 10 min. After a 5 min delay, two of the objects were relocated to new positions, and rats were allowed to explore for an additional 3 min. Exploration time at each object was recorded by an experimenter. The discrimination score was calculated as the percentage of time spent exploring the objects that had changed position vs. time spent on objects that remained in the same location [3].

### 2.4. In Vivo Electrophysiology

One day after completion of behavioral testing, representative rats from each group were randomly selected for in vivo electrophysiological recordings. Rats were anesthetized with chloral hydrate (400 mg/kg, i.p.) and maintained under anesthesia via intravenous (tail vein) administration as needed. Core body temperature was held at 37 ± 0.5 °C using a homeothermic blanket system (Harvard Apparatus, Holliston, MA, USA). Dopaminergic neuron activity was quantified using the “cells per track” method [41,42,43]. Nine electrode tracks were made on the right side of the VTA (coordinates from lambda: AP 2.7–3.3 mm; ML 0.5–0.9 mm; DV 6.5–8.5 mm), with 200 μm spacing between tracks. The final track in each animal was marked by iontophoretic ejection of pontamine sky blue dye (−20 μA for 10 min) and later confirmed by histological analysis.

### 2.5. Data Analysis and Statistics

Prior to statistical analysis, data normality was assessed using the D’Agostino and Pearson omnibus test for datasets with moderate to large sample sizes (*n* = 26−54 animals per group) and the Shapiro–Wilk test for smaller sample sizes (*n* = 12–15 animals per group). For normally distributed data, group comparisons (e.g., CD vs. WD; WD-Vehicle vs. WD-lorcaserin-betahistine) were performed using unpaired two-tailed t-tests with Welch’s correction to account for potential unequal variances. For non-normally distributed data, the Mann–Whitney U test was applied. Differences across multiple groups were assessed using a one-way ANOVA with Holm–Sidak post hoc testing for data that met normality assumptions or the Kruskal–Wallis test with Dunn’s post hoc comparisons for non-normally distributed data.

To assess the interaction between lorcaserin and betahistine in combination therapy, response additivity analysis was conducted [44,45]. The expected additive response for lorcaserin–betahistine was calculated as the average of the individual effects of lorcaserin and betahistine at corresponding doses. Observed responses for the combination therapy were compared to the expected additive response using a paired *t*-test. A *p*-value greater than 0.05 indicated an additive or synergistic interaction.

For the cells per track analysis, the number of dopaminergic and non-dopaminergic neurons recorded per track was averaged across all tracks for each animal to generate a single value per rat for statistical analysis. Only animals with successful recordings across all nine tracks were included in the final analysis, resulting in the following number of rats per group: CD-Vehicle (*n* = 14); WD-Vehicle (*n* = 12) and WD-lorcaserin + betahistine (*n* = 9). The firing rate of and variability in all recorded dopaminergic neurons included in the cells per track analysis were analyzed using commonly used and established protocols [38,43,46]. Bursting activity was identified using two methods: (1) the “80/160 ms” criterion, in which a burst was defined by two consecutive spikes with an interspike interval (ISI) of less than 80 ms to initiate the burst, and an ISI greater than 160 ms to mark its termination [47], and (2) the Poisson surprise method, where the threshold for burst detection varied based on the baseline firing rate, with each burst containing at least three spikes and a surprise value of ≥5 [46]. Data from individual cells were analyzed directly so that every recorded cell contributed equally to the calculated mean. Results from the above analyses are presented as the means ± S.E.M., except where otherwise indicated as mean ± S.D. Statistical significance was set at *p* < 0.05. Statistical analyses were performed using GraphPad Prism version 9.5 (GraphPad Software, San Diego, CA, USA).

## 3. Results

### 3.1. Effects of Western Diet (WD) on Body Weight and Morphometric Parameters

Following 8 weeks on the WD, rats showed a significant increase in body weight compared to those maintained on CD (Table 1, Figure S1a). In addition, multiple morphometric parameters, including body length, AC, thoracic circumference (TC), the AC/TC ratio, body mass index (BMI), and Lee index, were all significantly higher in the WD group relative to CD animals (Table 1).

### 3.2. Effects of WD on Learning and Memory Functions

**Figure 1:**
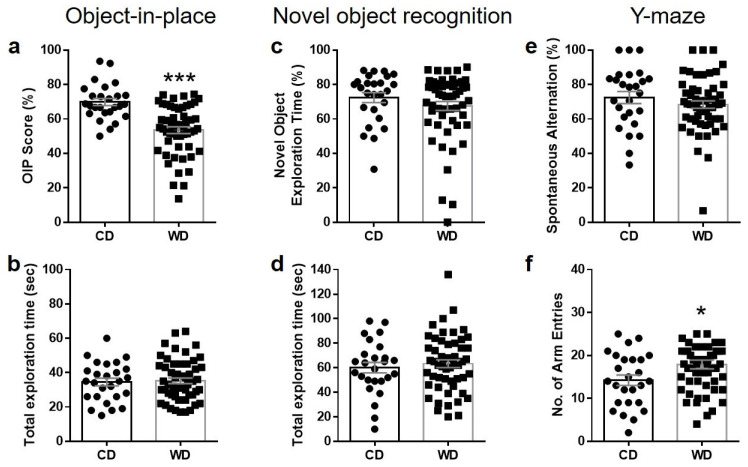
Impairments in object-in-place memory following chronic Western diet exposure. Rats fed a Western diet (WD) for 8 weeks showed significant deficits in the object-in-place (OIP) task compared to control diet (CD) rats (**a**), while performance in the novel object recognition (NOR) (**c**) and Y-maze tasks (**e**) were not altered. Total exploration times during OIP (**b**) and NOR tasks (**d**) did not differ between groups. In the Y-maze test, WD-fed rats exhibited a higher number of arm entries than CD rats (**f**). Data are presented as mean  ±  S.E.M. Each symbol denotes an individual animal in each group. *N* = 26 (CD) and 54 (WD). * *p*  <  0.05, *** *p*  <  0.001 vs. CD group.

Obese WD-fed rats showed significantly lower discrimination scores in the OIP task compared to CD-fed non-obese rats (t_66_ = 5.75, *p* < 0.001; Figure 1a). The total exploration time during the OIP task did not differ significantly between groups (t_52_ = 0.26; *p* = 0.39, Figure 1b). There were no significant differences in the performance on the NOR test (Mann–Whitney U = 614, *p* = 0.18; Figure 1c) or the spontaneous alternation Y-maze task (Mann–Whitney U = 613, *p* = 0.18; Figure 1e) between WD and CD groups. Exploration times for the NOR task were similar (t_52_ = 0.60, *p* = 0.79; Figure 1d), while the number of arm entries in the Y-maze was significantly higher in WD vs. CD rats (t_41_ = 1.85, *p* < 0.05; Figure 1f).

Given the robust sensitivity of the OIP task to WD-induced cognitive dysfunction, this test was selected for subsequent evaluation of the effects of lorcaserin and betahistine, alone or in combination.

### 3.3. Effects of Lorcaserin–Betahistine Combination on Object-in-Place Task Performance

**Figure 2:**
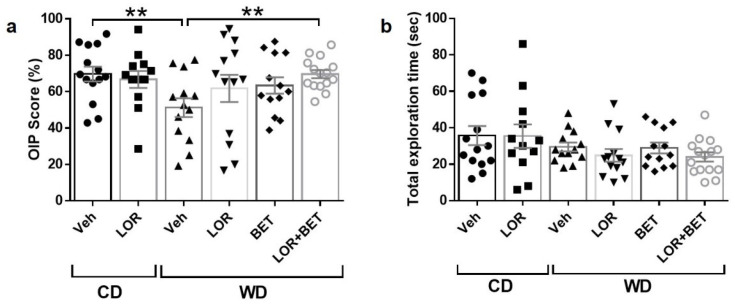
Effects of chronic lorcaserin–betahistine co-treatment on object-in-place memory in obese Western-diet-fed rats. (**a**) Thirty-day treatment of WD-fed rats with betahistine (BET; 5 mg/kg) or lorcaserin (LOR; 2 mg/kg) did not improve object-in-place (OIP) task performance compared to WD vehicle controls. However, co-administration of LOR + BET resulted in significantly higher OIP scores compared to vehicle-treated WD rats. (**b**) Total exploration time during the OIP task was not significantly affected by drug treatment. Data are presented as mean  ±  S.E.M. Each symbol denotes an individual animal in each group. *N* = 12–15 rats per group. ** *p* < 0.01.

After 30 days of treatment, vehicle-treated obese rats continued to show impaired OIP performance relative to CD rats (t_23_ = 2.83, *p* < 0.01; Figure 2a). A one-way ANOVA on OIP scores of WD rats revealed no statistically significant differences among the four groups (F_3, 50_ = 2.37, *p* = 0.08), indicating that, overall, the treatments did not produce a statistically robust effect across groups. However, because the *p*-value indicated a trend toward significance, we conducted further post testing and pairwise t-tests as exploratory analyses to further examine potential group differences. Holm–Sidak’s post hoc test identified a significant difference between vehicle-treated WD rats and lorcaserin–betahistine-administered WD obese animals (*p* < 0.05). This was corroborated by an unpaired t-test with Welch’s correction (t_16.4_ = 3.28, *p* < 0.01), suggesting a potential benefit of the combination treatment in this cohort. No other pairwise comparisons yielded statistically significant results (all *p*-values > 0.05). One-way ANOVA on the total exploration time in the OIP task of obese WD-fed rats revealed no statistically significant differences among the groups (F_5, 74_ = 1.56, *p* = 0.182, Figure 2b).

The response additivity analysis revealed that the observed response for lorcaserin–betahistine was not significantly different from the expected additive response (t_12_ = 1.52, *p* = 0.153; Figure S2). The mean difference between the observed and expected responses was 8.61%, suggesting that the combination therapy exerted an additive, rather than a synergistic or an antagonistic, effect.

Notably, none of the pharmacological treatments, either alone or in combination, produced significant changes in body weight or morphometric parameters in WD rats (compared to WD-vehicle controls) (Figure S1b; Table S1), indicating that cognitive improvements occurred independent of weight loss.

### 3.4. Effects of Combination Treatment on Dopaminergic Neuron Activity in the VTA

**Figure 3:**
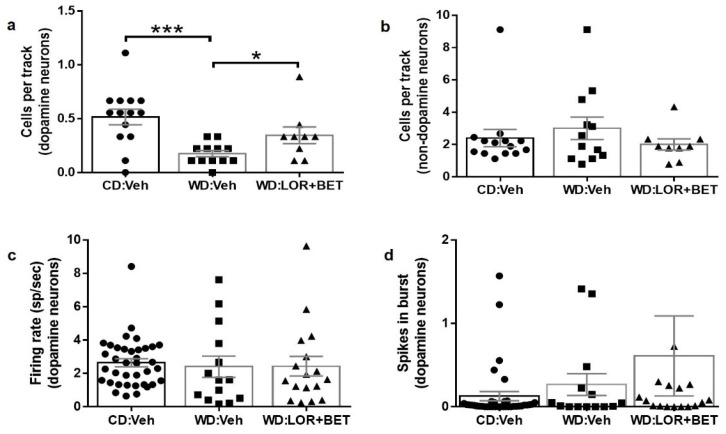
Effects of lorcaserin and betahistine co-treatment on ventral tegmental area (VTA) neuron activity in Western-diet-fed rats. (**a**) Western diet (WD)-fed rats showed reduction in the number of spontaneously active dopaminergic neurons in the VTA compared to control diet (CD) rats. Chronic co-administration of lorcaserin (LOR) and betahistine (BET) significantly increased the number of spontaneously active VTA dopaminergic neurons in WD rats. (**b**) The number of spontaneously active non-dopaminergic neurons in the VTA did not differ between WD rats treated with vehicle or LOR + BET. Data are presented as mean  ±  S.E.M. Each symbol represents the mean number of cells calculated across all tracks for each animal. *N* = 9–14 animals per group * *p* < 0.05, *** *p* < 0.001. (**c**,**d**) LOR + BET treatment did not alter the average firing rate (**c**) or firing pattern (**d**) of all recorded VTA-dopaminergic neurons. Data are presented as mean  ±  S.E.M. Each symbol represents one recorded cell.

Kruskal–Wallis tests revealed a significant overall difference among groups in the number of spontaneously active dopaminergic neurons in the VTA (H_2_ = 12.51, *p* < 0.01; Figure 3a). Dunn’s post hoc tests confirmed a significant reduction in spontaneously active dopaminergic neurons in obese WD-fed rats compared to non-obese CD-fed controls (*p* < 0.001) but did not detect a statistically significant difference between vehicle-treated WD controls and the LOR + BET group after correction for multiple comparisons. However, an unadjusted Mann–Whitney U test indicated a higher number of spontaneously active dopaminergic neurons in lorcaserin–betahistine-treated WD rats compared to vehicle-treated WD controls (Mann–Whitney U = 25, *p* < 0.05; Figure 3a).

For non-dopaminergic neurons, Kruskal–Wallis tests found no significant group effect (H_2_ = 0.465, *p* = 0.79; Figure 3b), and Mann–Whitney U tests did not identify statistically significant differences in any pairwise comparison. Similarly, Kruskal–Wallis tests showed that dopaminergic neuron firing rate (H_2_ = 2.471, *p* = 0.29; Figure 3c) and firing pattern (spikes in burst; H_2_ = 2.271, *p* = 0.32; Figure 3d) were comparable across groups. Subsequent post hoc and pairwise analyses further indicated that firing rate and firing pattern did not differ significantly between vehicle-treated CD- and WD-fed rat or between WD-vehicle and WD-lorcaserin-betahistine-treated animals.

## 4. Discussion

Obesity is now widely recognized as both a metabolic and neurocognitive disorder, with substantial evidence linking chronic exposure to high-fat, Western-style diets to long-term impairments in brain function, particularly within the PFC [4,5,8]. Consistent with prior findings, WD-fed rats in the present study showed an increase in body weight, obesity-associated morphometric profiles, and impairment in OIP task performance. Notably, chronic (30-day) combination lorcaserin–betahistine treatment significantly increased OIP scores and the number of spontaneously active dopaminergic neurons in the VTA of obese animals. These results indicate the therapeutic potential of lorcaserin–betahistine co-treatment and suggest that sustained enhancement in dopaminergic signaling may contribute to the amelioration of obesity-associated cognitive deficits.

In the present study, obese rats maintained on a WD exhibited deficits on a cognitive task requiring object–location discrimination, a function that engages multiple brain regions, particularly the PFC [3,48,49]. Interestingly, their performance in the Y-maze and novel object recognition tasks was not significantly altered, which was particularly notable for the Y-maze test as it also involves PFC-mediated functions [3,50]. However, we did observe an increased number of arm entries in obese rats in the Y-maze task, consistent with previous reports on effects of high-fat diet exposure in mice [51]. This selective cognitive impairment is consistent with the literature describing task-dependent variability in the cognitive effects of diet-induced obesity [52], which may be influenced by factors such as strain-specific differences (e.g., Long–Evans vs. Sprague-Dawley or Wistar rats), duration of dietary exposure (e.g., short-term 4-week vs. chronic 12-week high-fat feeding), and differences in the composition and fat content of the high-fat diet (e.g., 45% vs. 60% kcal from fat; lard-based vs. mixed fat sources).

Thirty days of lorcaserin or betahistine treatment did not improve OIP memory in WD-fed obese rats. In contrast, post hoc and pairwise analyses suggested that combined lorcaserin–betahistine treatment improved OIP scores in obese animals, ameliorating cognitive dysfunction to levels comparable to those of CD-fed animals. This finding is consistent with our hypothesis that lorcaserin and betahistine exert additive or synergistic effects by engaging distinct but complementary pharmacological targets to mitigate obesity-associated cognitive dysfunction [6]. Acutely, the drugs likely modulate dopaminergic signaling via activation or blockade of 5-HT_2_C (lorcaserin) and H_1_/H_3_ (betahistine) receptors [6]. Chronic treatment may lead to long-term neuroadaptations that enhance dopaminergic neuron activity, contributing to improved attention, learning, and memory. Notably, obese rats treated with the lorcaserin–betahistine combination showed an increased number of spontaneously active dopaminergic neurons compared to obese vehicle-treated rats, an important finding given the alterations in dopaminergic signaling within the mesocortical pathway associated with obesity [4,7,10,53]. Although the underlying mechanisms remain to be fully elucidated, they may involve drug-induced alterations in receptor expression or sensitivity (i.e., 5-HT_2_C and histamine receptors), leading to reduced inhibition or increased excitability of dopaminergic neurons. However, given the complex effects of 5-HT_2_C receptor activation on dopamine signaling [54] and the broader impact of H_3_ receptor blockade on multiple neurotransmitter systems aside from dopamine (i.e., acetylcholine, norepinephrine) [55], it is reasonable to speculate that the chronic effects of the drugs may also involve other neurotransmitters or signaling pathways. Additionally, an indirect mechanism may involve sustained increase in PFC dopamine levels induced by the drug combination, which could facilitate synaptic plasticity in this region [56,57,58] further contributing to the observed improvements in cognitive performance in WD-fed rats treated with both drugs. To further elucidate the mechanisms underlying the chronic effects of lorcaserin–betahistine, future studies should evaluate changes in dopamine release in the PFC using in vivo techniques such as microdialysis, fast-scan cyclic voltammetry, or fiber photometry. In parallel, molecular and biochemical analyses, such as quantitative PCR and Western blotting, could be used to assess expression levels of key genes and proteins involved in dopamine synthesis (e.g., tyrosine hydroxylase), receptor function (e.g., D1, D2, 5-HT_2_C, H_1_/H_3_), and dopamine transport (e.g., DAT, VMAT2) in the PFC and VTA. Moreover, the use of receptor-specific antagonists, such as SB242084 for 5-HT_2_C and triprolidine for H_1_ receptors, could determine the relative contributions of serotonergic and histaminergic pathways to the observed effects [6]. Investigating local circuit interactions in the VTA, including GABAergic and glutamatergic modulation, as well as upstream afferents from regions like the lateral hypothalamus or dorsal raphe, may also provide insight into how chronic lorcaserin–betahistine treatment activates the mesocortical pathway to ameliorate cognitive dysfunction. The current findings highlight the therapeutic potential of combinatorial, non-psychostimulant approaches for ameliorating obesity-associated neurocognitive dysfunction through long-term neuroadaptations. An additional advantage of this strategy is the reduced risk of abuse liability commonly associated with psychostimulant-based cognitive enhancers (e.g., phentermine, amphetamine) [6].

Notably, combination lorcaserin–betahistine treatment did not reduce body weight or morphometric measures in WD rats, indicating that the observed cognitive improvements in rats occurred independent of the drugs’ metabolic effects. This dissociation suggests that lorcaserin–betahistine selectively enhances PFC-associated cognitive function without influencing food intake or weight. Future studies should investigate whether metabolic markers such as insulin, glucose, or leptin resistance contribute to or are altered by combination therapy to further delineate the relationship between cognitive and metabolic effects of the drugs.

This study has some limitations that warrant consideration. First, while OIP performance served as the primary measure of cognitive function, the effects of lorcaserin–betahistine on other cognitive domains, such as attention or cognitive flexibility, were not evaluated. Future studies incorporating tasks that target these domains will provide a more comprehensive understanding of the combination treatment’s cognitive effects. Second, we employed subthreshold doses of each drug to assess potential additive or synergistic effects; however, dose–response studies are needed to fully characterize the complementary cognitive and neurochemical impact of the combination therapy. Moreover, the long-term behavioral effects of lorcaserin–betahistine remain unclear, including whether cognitive benefits are sustained over time or lead to improved self-regulation of food intake. Furthermore, this study did not examine individual variability in drug response, particularly sex differences, which may be crucial for both the generalizability and translational relevance of the findings. Lastly, this study used lorcaserin, which, as noted previously, was withdrawn from the U.S. market due to concerns about cancer risk. However, the insights gained from this study may inform the development of next-generation 5-HT_2_C receptor agonists with improved safety profiles, and their combination with histaminergic agents may selectively target cognitive deficits without inducing oncogenic risks.

## 5. Conclusions

This study demonstrated the potential efficacy of combined lorcaserin–betahistine treatment in ameliorating obesity-associated cognitive dysfunction in rats. The combination treatment improved performance on a PFC-associated behavior task and increased the number of spontaneously active VTA dopaminergic neuron in obese animals. These findings provide a preclinical basis for a novel, non-psychostimulant strategy to mitigate cognitive impairments linked to obesity, potentially via modulation of mesocortical dopaminergic pathways.

## Figures and Tables

**Table 1:** Effect of Western diet on weight and morphometric parameters in rats.

Parameters	Control Diet (CD)	Western Diet (WD)
Weight (g)	341.0 ± 36.36	403.1 ± 26.76 ***
Body length (cm)	24.55 ± 0.588	25.55 ± 0.755 ***
Abdominal circumference (cm)	16.16 ± 0.834	18.14 ± 0.815 ***
Thoracic circumference (cm)	15.02 ± 0.624	16.48 ± 0.602 ***
AC/TC	1.07 ± 0.036	1.10 ± 0.047 *
BMI (g/cm^2^)	0.56 ± 0.054	0.61 ± 0.039 ***
Lee Index	0.28 ± 0.009	0.29 ± 0.007 *

Values are mean ± standard deviation (S.D.) of the mean. BMI: body mass index; * *p* < 0.05; *** *p* < 0.001 significant vs. control diet rats.

## Data Availability

The data presented in this study are available from the corresponding author upon reasonable request because they are subject to institutional animal care ethics and confidentiality policies.

## Supplementary Materials

The following supporting information can be downloaded at: https://www.mdpi.com/article/10.3390/brainsci15090913/s1. Figure S1: Body weight changes in rats maintained on control or Western diet and following 30-day drug treatment. Figure S2: Response additivity analysis of observed vs. expected additive response for lorcaserin-betahistine (LOR+BET) combination treatment; Table S1: Effects of lorcaserin (LOR), betahistine (BET) and their combination on morphometric parameters in rats exposed to a Western Diet (WD) compared to Control Diet (CD).
