# Supplementary material for: Combination Lorcaserin and Betahistine Treatment Improves Cognitive Dysfunction and Dopaminergic Neuron Activity in a Rat Model of Diet-Induced Obesity
Source: Brain Sci. 2025 Aug 25;15(9):913. doi: 10.3390/brainsci15090913 (PMC12467822; doi:10.3390/brainsci15090913)
Supplement: Supplementary file 1 [file brainsci-15-00913-s001.zip › brainsci-3774434-supplementary.pdf]

Combination Lorcaserin and Betahistine Treatment Improves Cognitive Dysfunction and Dopaminergic Neuron Activity in a Rat Model of Diet-Induced Obesity

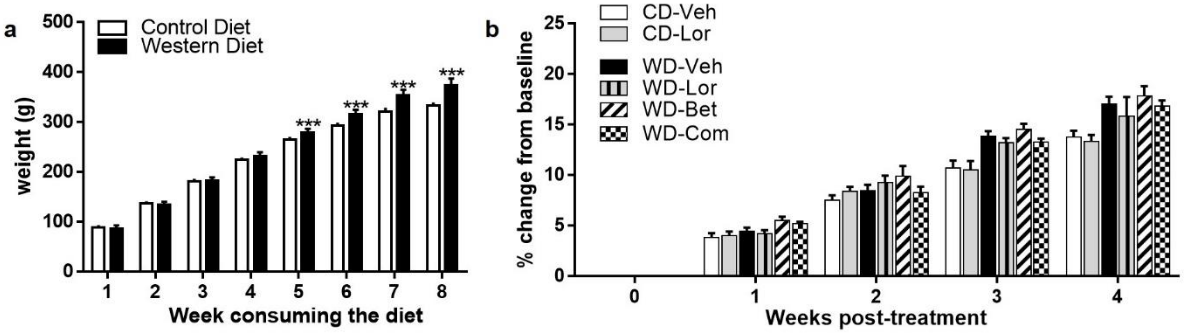

Supplementary Figure S1. Body weight changes in rats maintained on control or Western diet and following 30-day drug treatment. (a) Rats fed a Western diet (WD) for 8 weeks showed significantly increased body weight compared to control diet (CD) rats prior to drug treatment. Body weight data were recorded and analyzed only from representative, randomly selected animals from each group, as consistent weight changes were observed in the WD group based on previous findings from our laboratory [20-22]. (b) Change from baseline body weight differed between CD and WD rats receiving vehicle during the third and fourth weeks of treatment. However, chronic drug treatment did not significantly reduce body weight in WD-fed rats. Data are presented as mean ± S.E.M. ***$p < 0.001$ vs. CD group. Lor: lorcaserin; Bet: Betahistine; Com: Combination lorcaserin-betahistine treatment. n=12-15 animals per group.

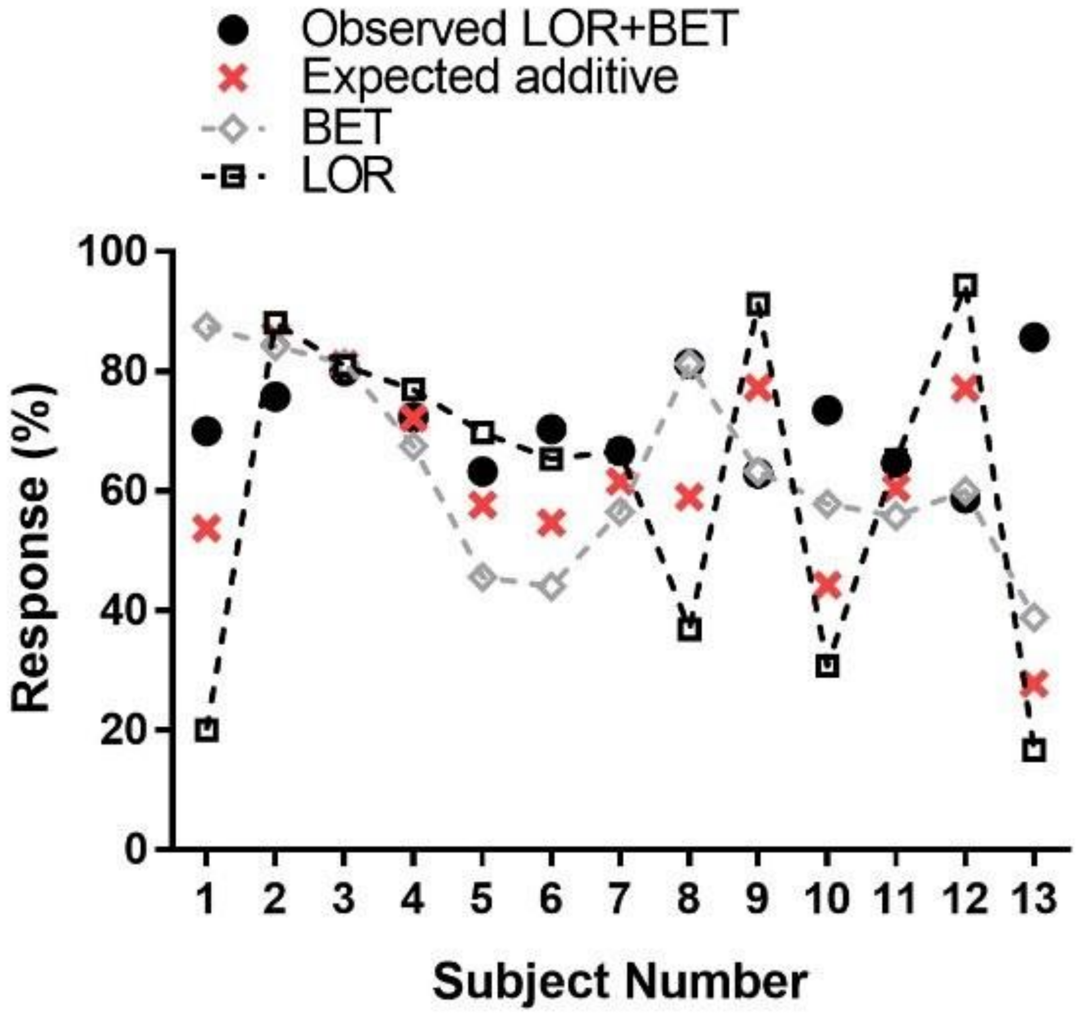

Supplementary Figure S2. Response additivity analysis of observed vs. expected additive response for lorcaserin-betahistine (LOR+BET) combination treatment. Data derived from object-in-place task performance. The plot shows the observed responses (black circles) for the combination therapy (LOR+BET) compared to the expected additive response (red "x" markers) calculated as the average of Lorcaserin (LOR) and Betahistine (BET) individual responses. The dashed lines represent the dose-response curves for Lorcaserin (LOR) and Betahistine (BET). Subject numbers (1 to 13) are shown on the x-axis, with the y-axis representing the response percentage. The comparison suggests that the

combination therapy exhibits an additive effect, as there was no significant difference between the observed and expected responses ($t_{12} = 1.52$, $p = 0.15$).

Table S1. Effects of lorcaserin (LOR), betahistine (BET) and their combination on morphometric parameters in rats exposed to a Western Diet (WD) compared to Control Diet (CD).

	**30 D post-treatment**					
	**CD**		**WD**			
**Parameters**	Veh	LOR	Veh	LOR	BET	LOR+BET
Weight (g)	396.8 ± 41.66	391.7 ± 33.65	470.1 ± 33.97**	457.6 ± 71.5*	490.4 ± 17.10***	475.7 ± 32.85***
Body length (cm)	25.75 ± 0.802	25.39 ± 1.163	26.38 ± 0.582	26.38 ± 0.443	27 ± 0.597**	26.75 ± 0.378
AC (cm)	16.89 ± 0.764	16.75 ± 0.580	19 ± 0.963***	19.44 ± 1.14***	19 ± 0.756***	18.94 ± 0.729***
TC (cm)	15.75 ± 0.643	15.89 ± 0.711	17.06 ± 0.563***	17.31 ± 0.704***	17.13 ± 0.443***	17.13 ± 0.640***
AC/TC	1.073 ± 0.037	1.055 ± 0.031	1.11 ± 0.036	1.122 ± 0.027*	1.10 ± 0.029	1.10 ± 0.035
BMI (g/cm^2)	0.59 ± 0.045	0.60 ± 0.020	0.67 ± 0.046*	0.66 ± 0.109	0.67 ± 0.033*	0.66 ± 0.032
Lee Index	0.28 ± 0.007	0.29 ±0.006	0.29 ± 0.007	0.29 ± 0.018	0.29 ± 0.006	0.29 ± 0.005

Values are mean ± standard deviation (S.D.) of the mean

AC: Abdominal circumference; TC: Thoracic circumference; BMI: Body mass index; **p<.05; **p<.01; ***p<0.001 significant vs. Control Diet rats;

Veh: Vehicle-treated rats
